# Reductions in co-contraction following neuromuscular re-education in people with knee osteoarthritis

**DOI:** 10.1186/s12891-016-1209-2

**Published:** 2016-08-27

**Authors:** Stephen J. Preece, Richard K. Jones, Christopher A. Brown, Timothy W. Cacciatore, Anthony K. P. Jones

**Affiliations:** 1 Centre for Health Sciences Research, University of Salford, Manchester, M6 6PU UK; 2 Human Pain Research Group, University of Manchester, Clinical Sciences Building, Salford Royal NHS Foundation Trust, Salford, M6 8HD UK; 3 Institute of Neurology, University College London, Queen Square, London, WC1N 3BG UK

**Keywords:** Knee osteoarthritis, Alexander Technique, Gait, Co-contraction, Pain, Electroencephalography

## Abstract

**Background:**

Both increased knee muscle co-contraction and alterations in central pain processing have been suggested to play a role in knee osteoarthritis pain. However, current interventions do not target either of these mechanisms. The Alexander Technique provides neuromuscular re-education and may also influence anticipation of pain. This study therefore sought to investigate the potential clinical effectiveness of the AT intervention in the management of knee osteoarthritis and also to identify a possible mechanism of action.

**Methods:**

A cohort of 21 participants with confirmed knee osteoarthritis were given 20 lessons of instruction in the Alexander Technique. In addition to clinical outcomes EMG data, quantifying knee muscle co-contraction and EEG data, characterising brain activity during anticipation of pain, were collected. All data were compared between baseline and post-intervention time points with a further 15-month clinical follow up. In addition, biomechanical data were collected from a healthy control group and compared with the data from the osteoarthritis subjects.

**Results:**

Following AT instruction the mean WOMAC pain score reduced by 56 % from 9.6 to 4.2 (*P* < 0.01) and this reduction was maintained at 15 month follow up. There was a clear decrease in medial co-contraction at the end of the intervention, towards the levels observed in the healthy control group, both during a pre-contact phase of gait (*p* < 0.05) and during early stance (*p* < 0.01). However, no changes in pain-anticipatory brain activity were observed. Interestingly, decreases in WOMAC pain were associated with reductions in medial co-contraction during the pre-contact phase of gait.

**Conclusions:**

This is the first study to investigate the potential effectiveness of an intervention aimed at increasing awareness of muscle behaviour in the clinical management of knee osteoarthritis. These data suggest a complex relationship between muscle contraction, joint loading and pain and support the idea that excessive muscle co-contraction may be a maladaptive response in this patient group. Furthermore, these data provide evidence that, if the activation of certain muscles can be reduced during gait, this may lead to positive long-term clinical outcomes. This finding challenges clinical management models of knee osteoarthritis which focus primarily on muscle strengthening.

**Trial registration:**

ISRCTN74086288, 4th January 2016, retrospectively registered.

## Background

Knee osteoarthritis (OA) is a major cause of disability and reduced quality of life across the world [1, 2] and has been estimated to affect over 12.5 % of the UK population [3]. Historically the condition was viewed as a degenerative disease of the articular cartilage and subchondral bone and that OA-related pain with a direct result of this destructive process. However, numerous studies have demonstrated a lack of concordance between radiographic measures of joint degeneration and clinical pain [4] suggesting that a range of different mechanisms may underlie knee OA pain [5] including peripheral and central mechanisms [6]. There is now a large body of evidence demonstrating that patients with knee OA exhibit excessive muscular co-contraction (simultaneous activation of the quadriceps and hamstrings) during walking [7–11] and other functional tasks [12–15]. This co-contraction increases compressive loads at the knee joint surface [16, 17], accelerates structural progression of the disease [18] and increases the likelihood that patients will progress to total knee arthroplasty [19]. Elevated loading may also increase the stress on articular structures, such as the joint, bone, synovium/joint capsule and periarticular structures, resulting in increased pain.

Another mechanism that has been suggested to mediate OA-related pain is an alteration in central pain perception, in which supraspinal processes affect nociceptive processing [20]. Support for this idea has been provided by experimental studies which have demonstrated an increase in anticipation-evoked EEG potentials in patients with OA [6]. These findings highlight the possibility that sensitisation of nociceptive pathways may result in patients with OA perceiving relatively low level stimuli as being overtly painful. Interestingly, a recent study demonstrated that it may be possible to reduce anticipatory pain activity with mindfulness training [21]. These findings demonstrate the possibility of using cognitive methods to influence supraspinal processing and potentially nociceptive signals.

Current conservative first-phase management of knee OA is primarily focused around physiotherapist-delivered exercise programmes. These programmes typically incorporate different components ranging from simple muscle strengthening/stretching [22–24] and aerobic walking [25, 26] through to balance and coordination training [27]. In a recent review it was concluded that the magnitude of the sustained (2–6 months) benefit from exercise programmes is small, with a typical reduction in pain of 6 points on a 0:100-point scale [28]. However, the mechanism of action of current exercise interventions is not clear. It is possible that these small improvements in pain could be the result of changes in muscular co-contraction or central pain processing. However, exercise interventions do not directly target these factors. Therefore further research is required to understand the potential clinical efficacy of interventions which have the potential to modify muscle activation patterns and/or central pain processing.

The Alexander Technique (AT) is a method of neuromuscular re-education which aims to teach individuals how to improve postural support, reduce potentially harmful patterns of muscle tension and improve control of response. AT lessons provide an individualised approach to developing skills that help people recognise, understand, and avoid poor habits adversely affecting postural tone and neuromuscular coordination. For further explanation of the AT, the reader is referred to the description provided by Cacciatore et al. [29].

Randomised controlled studies have shown beneficial effects of one-to-one AT lessons for a range of conditions [30]. For example AT lessons have been shown to improve clinical outcomes in people with chronic low back [31], reduce pain in individuals suffering with neck pain [32] and also to improve self-reported disability and depression in people diagnosed with Parkinson’s disease [33]. Research has also demonstrated that training in the AT can improve neuromuscular coordination and enhance the dynamic regulation of postural tone [34, 35]. In a recent study of the sit-to-stand movement, AT enabled smoother movement at lift-off which could be explained by reduced leg extensor resistance [36]. These findings suggest that improvements in neuromuscular control, which result from AT instruction, may include altered neuromuscular control of knee extensor moments. If this is the case then applying the AT may lead to reduced co-contraction of the knee muscles and therefore may be beneficial for patients with knee OA. As part of AT instruction, individuals are encouraged to become aware of, and consciously inhibit, increases in muscle activity which are triggered in anticipation of certain stimuli, such as those provoking pain. It is possible that this focus on anticipatory muscular behaviour could have an effect on supraspinal processes which influence nociceptive processing and therefore OA-related pain.

Given the potential of the AT to influence both muscular co-contraction and central pain processing, we designed a study to investigate potential mechanisms of action of the AT in the clinical management of knee OA. The first aim of the study was to develop an understanding of the magnitude of the clinical change, both in the short and long term, which may result from AT instruction. Secondly, we sought to understand if AT instruction would lead to reduced muscular co-contraction in a knee OA cohort towards the level characteristic of an age-matched healthy population, and also if it would alter anticipatory pain responses. The final aim of the study was to investigate relationships between changes in clinical outcomes and changes in co-contraction or anticipatory pain responses.

## Methods

In order to develop an understanding of the potential effect of the AT in knee OA, an uncontrolled pre-post design was used. With this approach a total of 21 participants with knee OA were assessed for pain, biomechanical function and pain processing before and after AT instruction. Further biomechanical comparisons were then made between the AT group and a cohort of age-matched healthy controls.

### Participants

A total of 22 participants with knee OA were recruited and offered AT lessons. As no previous data for the clinical effectiveness of AT lessons for knee OA were available, the sample size calculation was based on data from a study of a self-management and exercise rehabilitation programme [37]. This study reported a change in WOMAC pain scores of 1.5 SD immediately after the 20-sessson intervention. Based on these data, an a priori, within-sample calculation was performed using the g*power software. This showed that a target sample of *n* = 20 (assuming 10 % attrition), would be sufficient to detect a change of 0.75 SD in WOMAC pain score (half that reported in [37]) with a statistical power of 0.8 and an α = 0.05.

Participants with knee OA were recruited through four general practitioners in the Greater Manchester area. In each of the five practices, electronic patient records were reviewed to identify all participants who satisfied the following criteria:

1. X-ray diagnosis of knee OA.
2. Between 40 and 70 years of age.
3. No diabetes, systematic disorders, such as rheumatoid arthritis.
4. No lower limb arthroplasty.
5. No previous experience of the Alexander Technique.

A total of 150 letters of invitation were sent out. Those who replied (*n* = 38) underwent further telephone screening to ensure that they did not have any problems with balance, satisfied all the above criteria and regularly experienced knee pain during walking. A total of 22 individuals satisfied these criteria and all were invited to participate in the study. A healthy control group (*n* = 20) was also recruited in order to address the second research question, relating to biomechanical joint loading. This group was recruited via advert around the university and through local community groups, such as U3A and Rotary. Each healthy participant had to satisfy criteria 2–5 above and have no history of musculoskeletal disorders of the lower limb or spine. Healthy participants were selected so that the mean age and BMI of this group was matched to that of the knee OA group. Before testing all subjects provided written informed consent to participate in the study and ethical approval was obtained from the NRES Greater Manchester North ethics committee, reference: 11/NW/0057.

### The Alexander technique intervention

The Alexander Technique (AT) intervention was delivered by an experienced local practitioner who was a certified member of the Society of Teachers of the Alexander Technique. The AT is usually delivered on a one-to-one basis and, for this study, each participant was offered 20 one-to-one AT lessons each lasting 40 min. The lessons were delivered over a 12 week period, twice a week for the first 8 weeks and weekly for the final 4 weeks. This rate of delivery was chosen to ensure similarity with other clinical trials investigating the AT, as well as with routine practice [31, 32].

One of the primary objectives of AT instruction is to improve one’s overall pattern of postural muscle tension. This is achieved by guiding an individual to prioritise attention to maintaining a dynamic coordinated and lengthening central body axis and to improved awareness of appropriate and inappropriate postural and tensional patterns. Typically, these skills are taught using such common movements as sit-to-stand or walking, as well as with the person lying in a semi-supine position. The teacher uses gentle manual guidance and verbal instruction, together with constructive feedback, to enable the individual to lessen habitual interference with and maintain the lengthened axial tensional pattern, thereby reducing inappropriate tensional patterns in general, and typically leading to movement that perceivably requires less effort. In addition, they are taught to be aware of subtle increases in anticipatory muscle activity which are triggered during movement and also in anticipation of pain. Individuals are taught to interpose mental ‘directions’ between their intention to move and their execution of the movement so as to maintain postural support activity in the spine and torso, and to avoid overreacting in anticipation of the usual effort (or pain) involved. Following the guided sessions, participants were encouraged to continue to apply the skills learnt in the lessons as they went about their daily activities, maintaining an improved postural awareness and state and continuing to inhibit inappropriate tensional patterns.

### Clinical outcomes

Clinical effectiveness of the AT intervention was assessed using the WOMAC self-report questionnaire which captures information on pain, stiffness and function [38]. Given our hypothesis that AT instruction may lead to reduced co-contraction and subsequent pain, we defined the WOMAC pain score (5 items from the full WOMAC questionnaire) as the primary outcome measure. The full WOMAC score (20 items) was also analysed as a secondary outcome measure. Clinical outcomes were collected at baseline, immediately post intervention (within 1 week of the final AT lesson) and also at 15 months post baseline. In addition, a record of analgesia use during the week before the baseline assessment and the week before the post intervention assessment was taken as well as a record of any other therapy accessed during the intervention period.

### Biomechanical assessment and outcomes

Biomechanical data, used to characterise joint loading, and EEG data, used to characterise pain processing, were collected at baseline and immediately post intervention. Biomechanical data from a healthy cohort were also collected separately to characterise joint loading in this group. During each of the biomechanical testing sessions, participants walked barefoot at a speed of 1.25 ms^−1^ along a walkway. Speed was measured with optical timing gates and only speeds within ±10 % considered acceptable. All subjects apart from one participant with knee OA were able to walk at this speed. In this one participant a walking speed of 1 ms^−1^ was found to be appropriate and used for both the baseline and repeat testing. Although some previous studies of muscle activation, in people with knee OA, have instructed participants to walk at a self-selected speed [8], EMG amplitudes are known to vary with walking speed [39]. Therefore, in order to ensure appropriate comparison across testing sessions and between healthy and OA groups, we opted to control walking speed.

EMG data from the hamstrings and quadriceps was collected during walking to quantify co-contraction of the knee muscles. Data were collected using a Noraxon Telemyo system (3000Hz) with electrodes placed on vastus lateralis, vastus medialis, biceps femoris and semimembranosus according to SENIAM guidelines [40]. Following the walking trials, reference EMG data were collected during maximal voluntary isometric contractions (MVIC) for amplitude normalisation of the final EMG signals. To collect these data, the participant was seated in an isometric dynamometer with the knee flexed at 45° and, following a warm-up period, MVIC data collected first from the quadriceps and then the hamstrings. Three MVICs were performed for each muscle group with a 1 min rest between each contraction. During these contractions the net joint torque (measured with the dynamometer) was recorded in order to quantify knee extensor/flexor moments.

Following data collection, EMG data was exported to Matlab for processing. After applying a 20Hz high pass FFT filter to remove noise and movement artefact, the signal was rectified and then low pass filtered (6Hz Butterworth) to create a linear envelop [8]. The EMG signals were then time normalised to the stance period of the most affected limb using gait event data captured from the force platforms (see below). The MVIC data was filtered in the same way as the movement data and then a moving window algorithm used to determine the 0.1 s window in which the maximum EMG amplitude occurred [8]. This MVIC value was then used to normalise the EMG data from the walking trials. In order to characterise knee extensor/flexor strength, the peak torque was identified across the three maximal quadriceps/hamstring contractions.

We characterised muscular co-contraction during two specific phases of the gait cycle: an early stance phase and a pre-contact phase. Modelling studies have shown that, during early stance, there is a peak in the knee contact force which occurs between 15 and 25 % of stance [17]. It has also been shown that this peak increases with knee muscle co-contraction [17]. We therefore characterised muscular co-contraction during this period. We also characterised co-contraction over a pre-contact phase period (−5 % to 0 % of stance), just before initial contact. It has been shown that patients with knee OA increase background muscular co-contraction in anticipation of a destabilising perturbation [41] and it is possible that such anticipatory muscle activity may occur during walking, in preparation for contact with the ground. Such increased anticipatory contraction of the hamstring and quadriceps may lead to a subsequent increase in knee joint stiffness during the loading period. This may, in turn, affect the rate of increase of the knee contact force which could subsequently affect nociceptor input. As AT training aims to develop awareness and influence anticipatory muscle activity, it is possible that this intervention may influence co-contraction during the pre-contact phase.

A range of different algorithms have been proposed to calculate co-contraction of the knee muscles during walking. However, results from a recent modelling study suggested that simply summing the activity of the agonist and antagonist may give the best indication of articular loading [16]. Therefore, separate medial and lateral co-contraction EMG curves were obtained by summing the medial quadriceps and hamstrings and the lateral quadriceps and hamstrings respectively [10]. The final two co-contraction outcomes were then calculated as the respective means of the medial and lateral co-contraction curves over both the pre-contact phase (−5 % to 0 %) and the early stance phase (15 % to 25 %). Each time window was adjusted backwards to account for a 30 ms electromechanical delay.

Force and 3D motion plate data were also collected in order to quantify other aspects of joint loading. These data were collected using a 10-camera Qualisys Pro-reflex motion capture system (100 Hz) with two AMIT force plates (1500Hz) embedded in the walkway. Rigid clusters of 4 markers were used to track the motions of the thigh and shank, and a system of 4 markers, placed over anatomical landmarks, used to track motion of the foot [42]. Ankle and knee joint centres were calculated as midpoints between the malleoli and femoral epicondyles respectively and hip joint centres obtained using the regression model of Bell et al. [43]. Following data collection, the Visual 3D software (C-Motion, Rockville, Maryland) was used to derive the sagittal plane knee angle angles and also the sagittal and frontal plane knee moments from the kinematic data using a 6DOF model. These data were then time normalised to the stance phase. Peak sagittal angle, peak sagittal moment and peak frontal moment were then derived to characterise knee loading. Both peak moments were subsequently normalised to the participant’s body mass to define the final outcomes.

### Pain processing assessment and outcomes

We used a 64-channel EEG system (BrainAmp MR, BrainVision UK) to measure brain activity (sampling rate of 500Hz, FCz reference), and a thulium laser stimulator to induce 30 very brief (<150 ms) heat sensations on the right forearm. Prior to EEG recording, a suitable stimulus intensity was determined for each participant that induced moderately painful sensations. This was tailored to each individual patient using a psychophysics procedure, such that they judged a moderately pain sensation (described to the participant as clearly painful but easily tolerable) as a rating of 7 on a numerical rating scale (NRS) from 0 to 10. The resulting laser energy output (mean (standard deviation) across patients was 1.2 (0.3) Joules. During EEG recording, participants provided ratings of pain intensity for each laser pulse on the same 0 – 10 NRS. At 3 s, 2 s and 1 s prior to each stimulus onset, they were cued with an auditory stimulus to anticipate the timing of the pain, allowing for the recording of anticipatory brain responses. After each laser pulse, laser-evoked potentials were also recorded, allowing for measurement of the brain’s reaction to pain sensations. These anticipatory and laser-evoked responses were derived from the average, across all trials, of EEG responses time-locked to the stimulus onset, after pre-processing of the data using standard analysis techniques. These included filtering of the data to retain 0 – 30Hz frequencies, cleaning of eye-movement and other artefacts using Independent Components Analysis, manual rejection of remaining artefactual epochs, baseline correction to -3500 ms to –3000 ms pre-stimulus and re-referencing to the common average of all scalp channels.

**Fig. 1:**
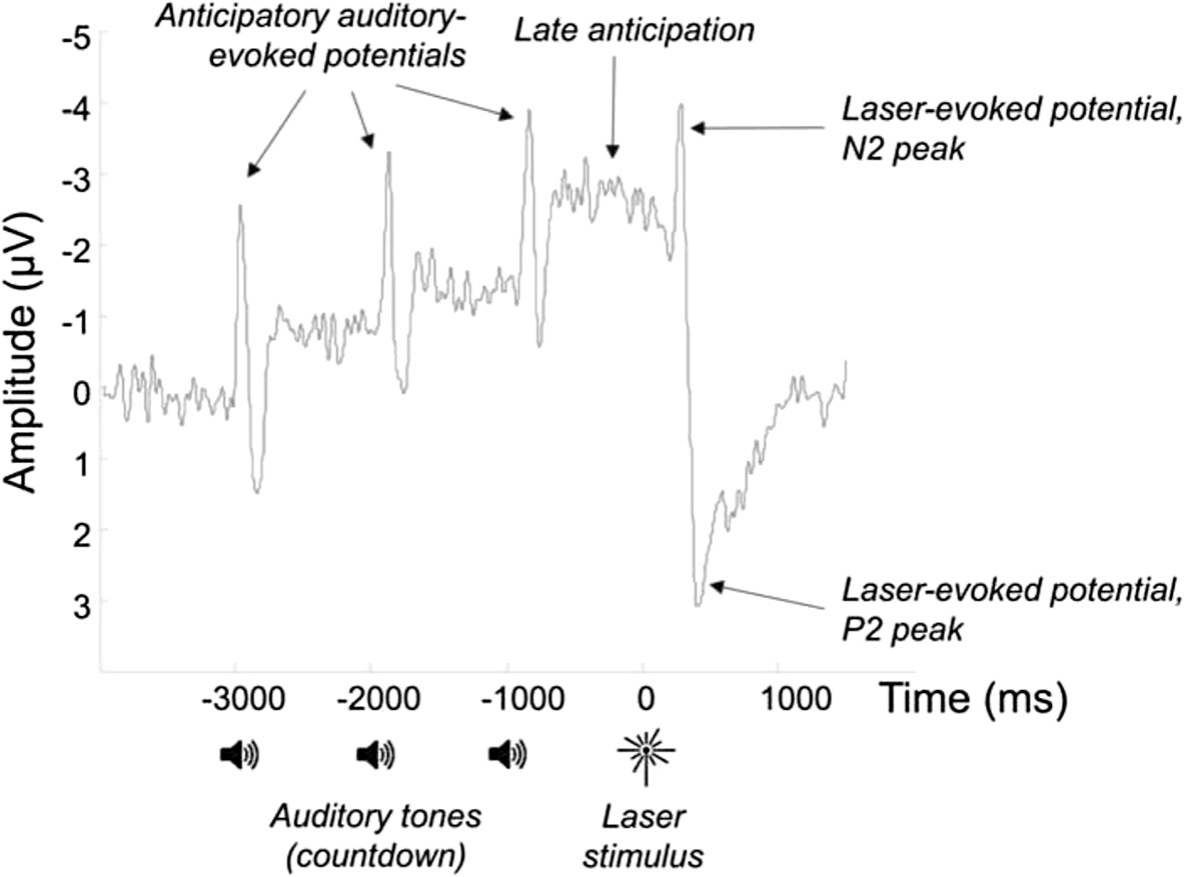
Anticipatory and laser-evoked potentials derived from the EEG signal, averaged across all participants with knee OA and testing sessions. Three auditory tones presented once per second counted down the onset of the laser stimulus

The analysis of the resulting event-related potentials (ERPs) focussed on two time windows. For anticipation, the mean amplitude of the ERP at electrode Cz and its eight surrounding electrodes during the late anticipatory period (−500 ms to 0 ms pre-stimulus – see Fig. 1) was analysed, as this previously showed enhanced amplitude in a study of OA patients relative to healthy controls [6]. To measure the neural response to pain, the relative difference between the N2 and P2 peaks of the laser-evoked potential was analysed, as these peaks have been previously shown to be modulated by cognitive factors such as attention and expectations of pain intensity [44, 45]. The N2 peak was specified as the largest negative deflection in the ERP in the time period between 200 ms and 350 ms after laser stimulation at electrode Cz, while P2 was the largest positive deflection between 350 ms and 500 ms at electrode Cz. For each peak, activity was averaged across electrode Cz and its eight surrounding electrodes over a 20 ms time window centred on the patients’ peak latencies.

### Statistical analysis

We used a Wilcoxon signed rank test to investigate changes in WOMAC pain and full WOMAC score following the AT intervention. Paired sample t-tests were used to establish whether any of the biomechanical or pain processing outcomes changed significantly following AT instruction. Following this, independent t-tests were used to compare the biomechanical loading variables between the healthy and knee OA groups. Finally, to investigate the link between changes in mechanistic outcomes and changes in clinical outcomes, we used a Pearson’s correlation analysis. For all analyses, α < 0.05 was chosen as the significance level. Although, with the relatively large number of statistical tests, this increases the likelihood of a type 1 error, it was deemed appropriate given the exploratory nature of this study.

## Results

### Participant disposition, adherence and baseline characteristics

A total of 22 individuals with knee OA were recruited and began the AT intervention. One participant dropped out (after 10 AT lessons) and was removed from the study; however the remaining 21 participants completed all 20 AT lessons. Biomechanical and clinical data was collected from all 21 participants at baseline and immediately post-intervention, however, only 19 participants agreed to undergo the pain processing assessment. Six participants were lost to long-term follow up, which left 15 patients with OA at the 15 month follow up.

There were no significant differences in baseline demographics between the healthy and OA groups (Table 1). The main WOMAC pain score of the knee OA participants was relatively high, reflecting our inclusion criteria of knee pain on walking. Kellgren and Lawrence (KL) [46] grades of the OA participants ranged from 2 to 4 with 11 participants being classified as KL = 2, nine participants as KL = 3 and four participants with KL = 4. Only six participants experienced unilateral symptoms with the rest reporting bilateral knee OA pain. Of the 21 participants, 15 reported other musculoskeletal pain, including shoulder, hip, neck and low back pain and 15 reported taking analgesia for their knee OA pain at baseline.

**Table 1:** Baseline demographics and disease characteristics for participants with knee OA and healthy control subjects

	Knee OA participants (*n* = 21)	Control subjects (*n* = 20)	*P*-value
Male/female	10/11	12/8	0.54
Age, years (SD)	62 (10)	61 (9)	0.68
Weight, kg (SD)	84 (13)	79 (14)	0.22
Height, cm SD)	169 (9)	170 (7)	0.8
BMI, kg/m^2^ (SD)	29 (4)	27 (4)	0.1
WOMAC pain (SD)	9.6 (3.0)	-	-
WOMAC overall (SD)	45 (13)	-	-

### Clinical outcomes

At the end of the AT instruction period there was a reduction from baseline of 56 % (*p* < 0.01) in the WOMAC pain score and 54 % (*p* < 0.01) in the full WOMAC score (Table 2). These reductions were maintained at 15 months (*p* < 0.01, Table 2). Of the 15 participants who had taken analgesia for their knee OA pain, 10 participants had reduced or stopped taking the medication and 5 had maintained the same level. Interestingly of the 15 participants reporting other musculoskeletal pain at baseline, 11 reported improvements in this non-knee related musculoskeletal pain immediately after the intervention. None of the participants accessed additional therapy during the intervention period.

**Table 2:** Clinical changes following AT instruction

	Change from baseline at end of intervention [SD] (N = 21)	Change from baseline at 15-month follow-up [SD] (N = 15)
WOMAC Pain Score	56 % (9.6 [3.0] - 4.2 [2.7]); *p* < 0.01	51 % (9.1 [3.2] to 4.4 [2.7]); *p* < 0.01
WOMAC Overall Score	54 % (45 [13] - 21 [13]); *p* < 0.01	43 % (43 [14] to 25 [14]); *p* < 0.01

### Biomechanical outcomes

Following AT instruction, medial co-contraction was observed to decrease by 13 % (*p* < 0.05) during the pre-contact phase, however, there was no significant change in lateral co-contraction during this period (*n* = 19). Note that, due to problems with the instrumentation, it was only possible to obtain EMG for 19 of the 21 OA participants. Comparison with the healthy controls showed medial co-contraction to be 39 % higher (*p* < 0.02) in the knee OA group at baseline but only 22 % higher following the AT intervention, a difference which was no longer significant (*p* = 0.12). There was no significant difference between the healthy controls and the OA group in lateral co-contraction either before or after the AT intervention.

**Fig. 2:**
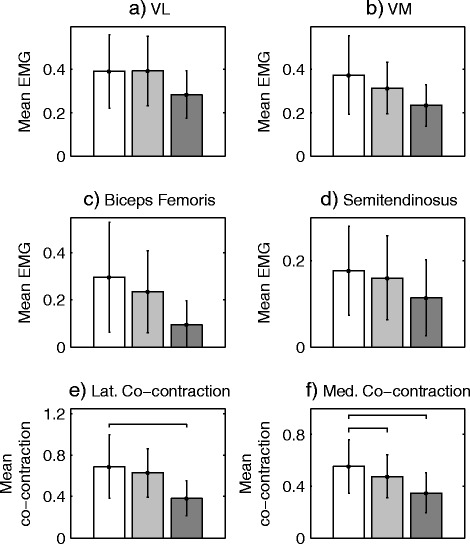
Mean normalised muscle activation during early the stance phase (15-25 %) for (**a**) vastus lateralis (VL), (**b**) vastus medialis (VM), (**c**) biceps femoris and (**d**) semitendinosus. Healthy participant data is shown in dark grey, baseline knee OA data in white and post-AT knee OA data in light grey. Plots (**e**) and (**f**) show lateral/medial co-contraction, calculated as the sum of hamstring and quadriceps activity and horizontal bars denotes significant differences (*p* < 0.01). Note that statistical testing was only performed on the measures of co-contraction

Similar changes in muscle activation were observed during the early-stance period following the AT intervention. Specifically, there was a reduction in medial hamstring (Fig. 2d) and medial quadriceps (Fig. 2b) activity which led to a 15 % reduction in medial co-contraction (*p* < 0.01, Fig. 2f). However the reduction in lateral co-contraction was not significant (*p* = 0.07, Fig. 2e). Comparison with the healthy controls showed medial co-contraction to be 57 % higher (*p* = 0.001) in the knee OA group at baseline. Although this reduced to 34 % following the intervention, the difference was still significant (*p* = 0.02). Analysis of the lateral co-contraction data revealed a similar pattern being 81 % higher (*P* < 0.001) in the OA group at baseline and 66 % (*p* < 0.001) higher following the AT intervention.

**Fig. 3:**
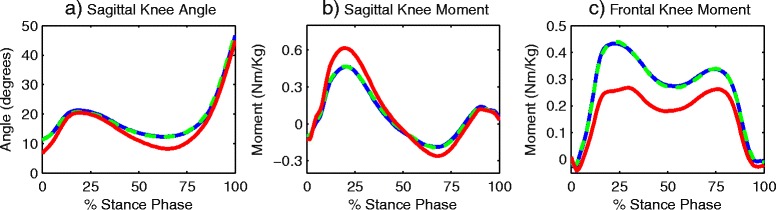
(**a**) Sagittal plane knee angle, (**b**) sagittal plane knee moment and (**c**) frontal plane knee moment for healthysubjects (red), OA patients at baseline (blue) and OA patients after the intervention (green)

The strength data, measured using the isokinetic dynamometer, showed no differences in either peak knee extensor torque (*p* = 0.72) or peak flexor torque (*p* = 0.27) following AT instruction. However, although healthy participants were able to generate peak knee extensor torques which were 47 % greater (*p* < 0.002) than those produced by the patients with knee OA, there were no group differences in peak knee flexor torque. A similar pattern was observed during walking, with no statistical changes in peak knee joint angle or peak moments (Fig. 3) following AT instruction. However, there was a significantly higher peak sagittal moment in the healthy subjects compared to the baseline OA data (*p* = 0.03) and a significantly higher (*p* < 0.01) peak knee adduction moment in the OA participants when compared to the control subjects.

### Pain processing outcomes

When the pain processing outcomes were analysed, no statistical changes were observed in either the late-anticipatory potential (*p* = 0.77) or the N2-P2 (Fig. 1) difference in the laser-evoked potential (*p* = 0.32).

### Relationship between biomechanical and clinical outcomes

**Fig. 4:**
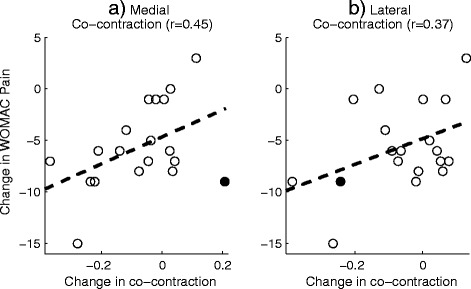
The relationship between the change in WOMAC pain (following the intervention) and the change in **a**) lateral and **b**) medial co-contraction during the pre-contact phase. The filled circle shows the outlying data point

Correlational analyses were carried out to test for a relationship between the change in WOMAC pain score and the change in medial/lateral co-contraction following AT instruction. These analyses were performed separately for the two different phases: the pre-contact phase and the early stance phase. For the pre-contact phase, a moderate correlation (*r* = 0.45, *p* < 0.05) was observed between the change in the WOMAC pain score and the change in medial co-contraction (Fig. 4a). This relationship showed a clear outlier (Fig. 4a), which when excluded, increased the correlation to *r* = 0.63, *p* < 0.01. There was some evidence of a correlation between the change in lateral co-contraction and change in the WOMAC pain during the pre-contact period, however this failed to reach significance (*r* = 0.37, *p* = 0.13, Fig. 4b). Furthermore, there was no evidence of a correlation between either of the co-contraction measures during the early stance period nor were any meaningful correlations observed between the changes in pain processing outcomes and the change in WOMAC pain.

## Discussion

We hypothesised that instruction in the AT may influence both muscular co-contraction and central pain processing and that, via these mechanisms, there would be an improvement in clinical pain and function in people with knee OA. Although this was not a randomised controlled trial, the data support the idea that AT may be an effective clinical intervention for people with knee OA and that it may reduce medial co-contraction. However, we did not find evidence that AT lessons influenced central pain processing. Our original hypothesis for investigating anticipatory brain activity was based on a study which showed that mindfulness may alter EEG activity triggered in anticipation of pain [21]. However, although this study did show some evidence that the AT may influence anticipatory muscle activity during walking, there was no evidence that this led to a concomitant reduction in either anticipatory, or pain-evoked, EEG activity resulting from experimentally induced pain at standardised intensities. These finding may indicate that the effects of AT are mainly related to muscle function and less likely to be due to cognitive effects on early anticipatory processing of pain that have been seen with other interventions such as placebo and mindfulness-based CBT. However, it is possible that the AT does influence pain processing via mechanisms which were not captured using the EEG outcomes measured in this study or that the null finding was a result of the relatively low sample size. Nevertheless, the results motivate further study into interventions for people with knee OA which can decrease muscle co-contraction.

To our knowledge, this is the first study to investigate the potential effectiveness of an intervention which is aimed at increasing awareness of muscle activation patterns for the clinical management of knee OA. The results are very encouraging and compare favourably with the results of previous trials which have investigated exercise-based management approaches [28]. The present study did not include a control group receiving usual care, however large scale RCTs of knee OA typically show reductions of approximately 1.5 points in WOMAC pain in a usual care arm over a 6–18 month period [37]. Our data showed a reduction of approximately 5 points in our intervention cohort over a 3 month period which appeared to be maintained 12 months after the end of the intervention. This is considerably larger than the 2.5-point reduction in WOMAC pain typical of exercise-based interventions [37]. These results identify the need for further large-scale trials to confirm whether lessons in the AT can bring about long-term improvement in pain and function in people with knee OA.

Given the potential implications for disease progression [18], there has been considerable interest in conservative interventions which have the potential to reduce co-contraction. For example, research into knee bracing has consistently shown that this approach can reduce co-contraction, in some cases by up to 35 % [47]. However, compliance is problematic and bracing may not be a viable long-term option for many patients with knee OA. Studies of exercise interventions provide conflicting findings, with some showing no change [48] and others a decrease in co-contraction [49]. Again, long-term compliance to muscle strengthening programmes can be poor [50]. Furthermore, it is not clear whether reductions in co-contraction, associated with strengthening programmes, are the result of an increase in the MVIC value used to normalise the gait EMG measurement. Interestingly, our data did not demonstrate any changes in muscle strength following the AT intervention, and this illustrates that substantial improvements in pain and function are possible without increases in strength. This finding challenges current clinical management models of knee OA which focus primarily on muscle strengthening.

Co-contraction has been shown to increase compressive loads at the knee joint surface [16, 17] and increase the likelihood that a patient will opt for a knee replacement at 5 year follow up [19]. In a recent study medial, but not lateral, co-contraction was found to speed up the rate of cartilage loss in people with knee OA [18]. In line with this finding, our data showed that AT lessons led to reduced medial co-contraction during the early stance phase, a period when joint contact forces are near maximal [17]. This finding suggests that AT instruction could reduce the articular loads on the knee joint and this may have a long-term protective effect, reducing the rate of joint destruction. Interestingly, we did not observe any changes in knee kinematics or kinetics following AT instruction which indicates that, although AT lessons led to reduced co-contraction, the net moments generated at the knee joint remained constant.

Although this study included participants with a range of KL grades, it was not powered to detect clinical or mechanistic differences in treatment response between different levels of disease severity. It is conceivable that individuals with less severe knee OA (lower KL grade) may derive more clinical benefit from interventions, such as the AT, which have the potential to decrease co-contraction. However, previous research has shown that co-contraction is consistently observed in individuals with knee OA, irrespective of the level of disease severity [51]. Furthermore, there is no clear link between clinical pain and the level of radiographic degeneration. Therefore, it may be the magnitude of the change in medial co-contraction which may dictate the clinical benefit, rather than the severity of the disease. However, appropriately powered controlled studies are required to confirm this idea.

The data from this study support the idea of a link between a reduction in medial co-contraction and an improvement in clinical pain (Fig. 4a). However, this link was only observed when muscle activation was characterised during the pre-contact phase and not during the early stance phase of gait. This finding suggests that it may not be the net magnitude of the peak knee loads which dictates pain. Instead, there may be a more complex relationship between preparatory muscle activity, influencing active joint stiffness and rate of knee loading and the subsequent nociceptor response and perceived pain. It is has been suggested that co-contraction may be a coping response to counteract perceived knee joint instability [41], and our data may indicate that this increase in muscle activation occurs immediately before contact is made with the ground. However, there is currently debate as to whether this response should be counteracted [52]. The findings that larger reductions in clinical pain were associated with more reduction in co-contraction (Fig. 4b) supports the idea of co-contraction as a maladaptive response and suggests that medial co-contraction may prove to be an effective treatment target in people with knee OA.

There were a number of limitations to this study which should be highlighted. Firstly, as the study was exploratory in nature, we did not include a patient control group. However, biomechanical data were collected data from a healthy control group allowing us to demonstrate that AT lessons led to reductions in medial co-contraction which were more in line with those observed in healthy individuals. Another limitation was that all AT lessons were delivered by a single AT practitioner and so it is not clear whether the large clinical effects observed in this study would be achieved consistently across all AT practitioners. Further large scale trials are needed to address this issue. In addition, we did not explore changes in physical activity patterns which occurred during the intervention period. However, although it is possible that changes in activity patterns influenced the observed clinical benefit, it is also conceivable that reductions in pain may lead to increases in physical activity. Finally, although we quantified muscle co-contraction, we did not use a modelling approach to precisely quantify peak contact forces at the knee. This was deemed to be beyond the scope of this study. However, our results suggest that the relationship between joint loading, muscle contraction and pain may be highly complex. Therefore, despite the omission of complex modelling techniques, we feel this study motivates new enquiry into the mechanism of pain in knee OA populations.

## Conclusion

This study was carried out to understand the potential clinical effectiveness of the AT in the management of knee OA and also to discriminate between different potential mechanisms of therapeutic action. Following AT instruction, there was a significant reduction in knee pain and stiffness and an improvement in function which appeared to be maintained at 15 months post-baseline. The mechanistic data supported the idea that clinical changes in pain were correlated with muscular co-contraction but we did not find evidence that the AT altered central pain processing. These findings suggest reduced medial co-contraction to be a potential mechanism for improvements in pain following 12 weeks of AT. Although further research is required to fully confirm these findings, this study demonstrates the potential efficacy of interventions, such as the AT, which can successfully modify muscle activation patterns in patients with knee OA.

## Abbreviations

OA: Osteoarthritis
AT: Alexander Technique
EMG: Electromyography
EEG: Electroencephalogram
